# Advanced Echocardiography in Adult Zebrafish Reveals Delayed Recovery of Heart Function after Myocardial Cryoinjury

**DOI:** 10.1371/journal.pone.0122665

**Published:** 2015-04-08

**Authors:** Selina J. Hein, Lorenz H. Lehmann, Mandy Kossack, Lonny Juergensen, Dieter Fuchs, Hugo A. Katus, David Hassel

**Affiliations:** 1 Department of Medicine III, Cardiology, Heidelberg University Hospital, 69120 Heidelberg, Germany and DZHK (German Center for Cardiovascular Research), Partner Site Heidelberg/Mannheim, Heidelberg, Germany; 2 FUJIFILM VisualSonics Inc., Amsterdam, The Netherlands; The University of Queensland, AUSTRALIA

## Abstract

Translucent zebrafish larvae represent an established model to analyze genetics of cardiac development and human cardiac disease. More recently adult zebrafish are utilized to evaluate mechanisms of cardiac regeneration and by benefiting from recent genome editing technologies, including TALEN and CRISPR, adult zebrafish are emerging as a valuable in vivo model to evaluate novel disease genes and specifically validate disease causing mutations and their underlying pathomechanisms. However, methods to sensitively and non-invasively assess cardiac morphology and performance in adult zebrafish are still limited. We here present a standardized examination protocol to broadly assess cardiac performance in adult zebrafish by advancing conventional echocardiography with modern speckle-tracking analyses. This allows accurate detection of changes in cardiac performance and further enables highly sensitive assessment of regional myocardial motion and deformation in high spatio-temporal resolution. Combining conventional echocardiography measurements with radial and longitudinal velocity, displacement, strain, strain rate and myocardial wall delay rates after myocardial cryoinjury permitted to non-invasively determine injury dimensions and to longitudinally follow functional recovery during cardiac regeneration. We show that functional recovery of cryoinjured hearts occurs in three distinct phases. Importantly, the regeneration process after cryoinjury extends far beyond the proposed 45 days described for ventricular resection with reconstitution of myocardial performance up to 180 days post-injury (dpi). The imaging modalities evaluated here allow sensitive cardiac phenotyping and contribute to further establish adult zebrafish as valuable cardiac disease model beyond the larval developmental stage.

## Introduction

Zebrafish represents an established in vivo model to study cardiovascular development and function [1]. Large forward genetic screens and extensive reverse genetics approaches yielded hundreds of novel genes essential for cardiovascular development and function and identified several novel disease genes and mechanisms for human heart failure [2–5]. Further, through recent progress in targeted genome editing by TALEN or CRISPR technology, zebrafish serves as a valuable in vivo model to evaluate and identify novel disease genes and validate disease causing mutations and their underlying pathomechanism [6–8]. Due to its translucency during larval stages evaluating cardiac morphology and measuring cardiac function in zebrafish embryos is easily achievable using common light microscopy. Cardiac disease in humans though typically affects elderly subjects and even carriers of disease genes remain phenotypically unremarkable during childhood and in younger adults. Importantly, examples for zebrafish models of adulthood manifestation of cardiomyopathy phenotypes exist and would benefit from detailed analysis of cardiac function [9–11]. Further, adult zebrafish are commonly deployed to study molecular mechanisms driving cardiac regeneration after myocardial injury [12–18]. However, analyzing cardiac form and performance during adult stages where translucency is lost remains technically challenging. Thereby, methods to non-invasively assess cardiac form and function in great detail would be of great value and would enable the establishment of zebrafish as a cardiac disease model beyond the larval stages.

Several imaging modalities have been proposed so far to evaluate cardiac form and function in adult zebrafish, including magnetic resonance imaging (MRI), echocardiography, optical coherence tomography and fluorescent light microcopy [19–22]. In humans and mice, the standard technique to non-invasively evaluate heart function is echocardiography, as it presents a relatively cost effective, easily commercially available technology with no exposure to radiation, short examination duration, high temporal resolution and relatively fast acquisition time. To benefit from these advantages in basic research, commercially available echocardiography devices, technically adapted to small animals’ dimensions, have been developed. A few studies exist that deployed echocardiography in adult zebrafish to monitor cardiac performance, including during heart regeneration [21, 23–27]. However, echocardiography in adult zebrafish was never applied following regeneration in response to cryocauterization, shown to best resemble the conditions in the mammalian heart after myocardial infarction [28–30]. Further, previous studies limited their analyses on global cardiac performance parameters without including modern methods to assess regional myocardial motion and deformation mechanics by speckle-tracking algorithms [31]. Speckle-tracking based strain analysis is a novel technic used in echocardiography to unmask regional cardiac deformation deficiencies. It permits detection of subtle changes in cardiac mechanics allowing highly sensitive detection of discrete dysfunctional myocardial regions in high spatio-temporal resolution as recently introduced in humans and mice [31–34].

Ultrasound naturally generates interferences called speckle-noise in tissues. Interestingly, the speckle noise follows the myocardial movement. Furthermore, every region in the myocardial wall creates its individual speckle pattern, enabling assessment of the movement of multiple myocardial regions during cardiac activity in very high spatial resolution [35]. The movement of every spot is calculated and mathematically assigned to a dynamic vector (in adult zebrafish hearts, see S4 Movie). Speckle-tracking thereby allows calculation of velocity and displacement for each speckle, and by putting at least two regions in relation, determining strain and strain rate [36].

Here we present an advanced protocol to globally and regionally evaluate cardiac performance in adult zebrafish non-invasively. In accordance to clinical practice in humans, we established three defined imaging planes specifically adapted to the anatomic characteristics of zebrafish using a commercially available echocardiography device. We assessed circumferential shortening parameters in the transversal short axis (SAX), longitudinal fractional shortening, ejection fraction and stroke volume measurements in the sagittal long axis (LAX) and pulsed-wave Doppler measurements in the abdominal-cranial axis (ACX). With this we were able to consistently and sensitively record physiological cardiac parameters in the adult zebrafish. Further, we detected distinct changes in cardiac performance in response to adrenergic stimulation or inhibition by pharmacological treatment. By applying a combination of conventional echocardiography with radial and longitudinal strain, strain rate and myocardial wall delay rate analyses, we were able to longitudinally follow cardiac regeneration after cryoinjury in adult zebrafish. While previous studies indicated that structural and functional recovery after ventricular resection is completed by around 45 days after injury, our results indicate an extended functional healing process after cryoinjury with three discriminable phases, which extends at least up to 6 months post-injury.

## Methods

All animal experiments have been performed in accordance with the guidelines of the state of Baden-Wuerttemberg and have been approved by the Regierungspräsidium Karlsruhe (permit number 35–9185.81/G-62).

### Zebrafish care and breeding

Care and breeding of zebrafish (Danio rerio) was performed essentially as described previously [37]. For all experiments we used 3–6 month old wildtype TE and *Tg(myl7*:*GFP)* ^*f1*^ adult zebrafish [38]. All animal experiments were conducted with approval of local Animal Care Committee and according to institutional guidelines.

### Cryoinjury

In adult zebrafish cryoinjury was induced as previously described [28–30]. In brief, adult zebrafish were transferred to anesthesia medium containing 2-Phenoxyethanol (Sigma-Aldrich) in a 1:2000 dilution to a final concentration of 3.2μM, known to not affect heart function [21, 27, 39]. After corpus and operculum movement have declined, the zebrafish were placed ventral side up on a sponge with a central incision to softly fix the corpus in the vertical position. Using a microscope (Olympus SCX 16) the thoracic area was opened through a 2mm incision at the median line between the two gills using high precision scissors. After incision of the skin and muscle the silver epithelial layer of the hypodermis was removed and the ventricle became well visible. Afterwards the cryoprobe, precooled for at least three minutes in liquid nitrogen, with a 0.8mm diameter at its tip is placed at the anterior myocardial wall for 22 seconds. Atraumatic detachment was achieved by rinsing the frozen area with fresh fish water. By pressing the two wound margins together, the body cavity was closed. Subsequently, the zebrafish was transferred to fresh water. Fish usually recovered within 15 seconds from narcosis. Maximal time in narcosis for each fish was approximately 150 seconds. Generally adult zebrafish at a minimum age of 3 months raised in a 20 l tank with a population density of 20–40 animals per tank satisfyingly survive the procedure.

Cardiac performance was serially evaluated at baseline, 1 day after injury (dpi), 4dpi, 7dpi, 14dpi, 30dpi, 60dpi and 120dpi. For beta-adrenergic treatment we added Atenolol (Sigma-Aldrich) or Isoproterenol (Sigma-Aldrich) to a final concentration of 100μM to the fish water. Echocardiographic examination was conducted 24 hours after treatment.

### Echocardiography

All zebrafish echocardiography measurements were performed using the Vevo2100 Imaging System including Vevo Imaging Station (VisualSonics, Amsterdam, Netherlands) equipped with high frequency transducer (MS550D, 22-55MHz) combined with the software provided by the manufacturer (VevoStrain Analysis software package, Version 1.5.0). Researchers establishing zebrafish echocardiography in their laboratories should be familiar with zebrafish anatomy and, as with every complex technical equipment and method, should be trained in the handling of the utilized echocardiography device. Since we used the Vevo2100 Ultrasound System combined with the software provided by the manufacturer, for more detailed information about the device as well as employed calculation algorithms, we refer to its official open access user manual [40]. Echocardiography was performed under anesthesia with 2-Phenoxyethanol (Sigma-Aldrich) as described above. After corpus and operculum movements declined, we placed the zebrafish, abdominal side facing upwards, on a sponge with a central incision forming a cavity to softly fixate the back and corpus in the vertical position. The corpus was covered with a thin plastic sheet to prevent conglutination of the gills by the ultrasound gel (Dahlhausen, Köln, Germany). The examination was conducted after transference to the Vevo Imaging Station (VisualSonics, Amsterdam, Netherlands), allowing precise and stable transducer positioning. After the recording of the three planes the zebrafish was carefully placed into a fish tank filled with fresh water. The zebrafish usually recovered within 15 seconds.

**Fig 1:**
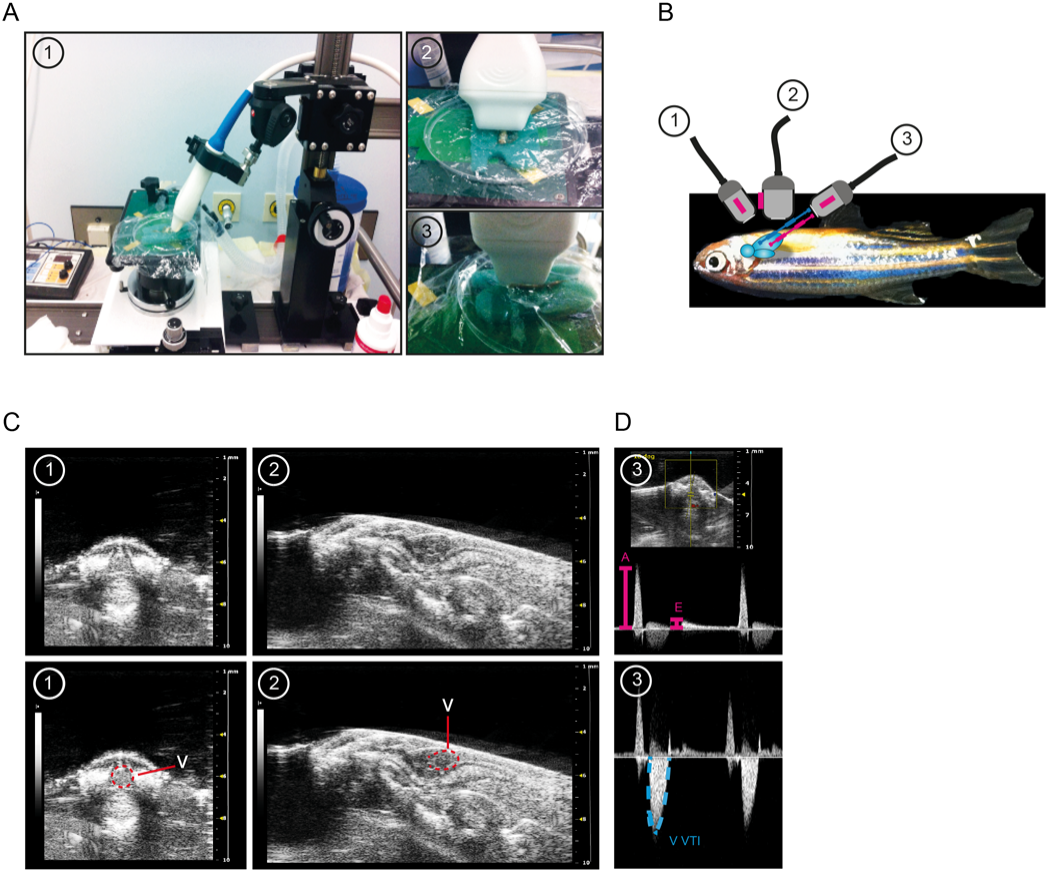
Three plane echocardiography to assess heart function in adult zebrafish. (**A**) Overview of experimental setting and illustration of transducer positioning to image short axis view (SAX) (1), abdominal-cranial axis (ACX) for pulsed-wave Doppler (PWD) recordings (2), and long axis view (LAX) (3). (**B**) Lateral view of dorsally positioned adult zebrafish with three defined transducer positions illustrated. The red line indicates direction of ultrasound beam for PWD acquisition for cardiac inflow velocity imaging and the blue line indicates ultrasound beam direction for acquisition of cardiac outflow velocities. (**C**) Representative images of SAX view (1) and LAX view (2) acquisition (upper row). In the lower row the ventricle is outlined in red in (1) and (2). (**D**) PWD images derived from the ACX view (3) with the upper image showing representative signals attained from the AV-valve region with clearly visible positive A- and E- waves (in pink) and the bottom image displaying one representative V VTI PWD signal obtained from the bulbus arteriosus region. PWD, pulsed-wave Doppler.

To acquire short axis view (SAX) recordings the transducer was positioned in a 60° angle onto the zebrafish corpus starting from the cranial side and moving caudal until ventricular basis was reached and ventricular boundaries were detected as a round shape with circular contraction in its maximum extension (Fig 1A1 and 1B1). B-Mode imaging quality was optimized by adaption of focus depth, 2D gain, image width and depth according to the manufacturer’s recommendations. From each plane 2–3 representative image sequences were recorded. The examination table was then rotated 180° and the ultrasound beam was directed in a 45° angle towards the abdominal wall to obtain the abdominocranial view (ACX) using the B-Mode imaging (Fig 1A3 and 1B3). The correct image plane for the ACX view is characterized by a clear view onto the u-shaped ventricle and valvular plane located in the cranial direction. In a second step C-Mode imaging was conducted to locate the areas with maximal flow velocities by stepwise augmentation of the Doppler gain. Maximum cardiac inflow velocity was usually located at the tip leaflets of the atrioventricular valve. The pulsed-wave Doppler (PWD) sample volume was placed at the region of maximum inflow velocity identified using C-Mode imaging and the PWD signal was detected for at least three seconds. To attain the maximum PWD outflow signals the PWD sample was placed at the conus arteriosus adjacent to the bulboventricular valve after C-Mode imaging guided identification of maximal outflow velocities. Therefore, transducer position needed to be shifted 35–50 μm (depending on the animals size) to a more cranial plane displaying the upper thoracic aperture, as illustrated in Fig 1B3 by the blue line. Similar to recording of inflow PWD signaling, outflow PWD signals were recorded for at least three seconds. Finally the experimental platform was turned 90° to attain long axis (LAX) view. Now the transducer was placed strictly vertically at the median line of the zebrafish corpus along his longitudinal axis (Fig 1A2 and 1B2). After image optimization, equally as described for SAX, at least two B-Mode image sequences capturing 4–5 cardiac cycles were recorded. Repeated measurements deploying our method on four animals on three consecutive days assessing systolic and diastolic area revealed highly consistent and highly reproducible measurements with an interclass correlation coefficient (ICC) of 0.98 and 0.99, respectively.

Data analysis was conducted off-line on a VisualSonics workstation. From the SAX the fractional area change (FAC) was calculated using the LV Trace analysis tool according to manufacturer’s manual. LV Trace was used similarly in LAX. Fractional shortening (FS), stroke volume (SV) and ejection fraction (EF) were calculated from systolic and diastolic ventricular area. From PWD signals heart frequency was calculated. Furthermore, from cardiac outflow velocities the ventricular velocity time integral (V VTI) was calculated and from cardiac inflow velocities maximal velocity of A- and E wave and the artrioventricular velocity time integral (AV VTI) were derived.

Speckle-tracking was conducted using LAX and VevoStrain Analysis software package (Version 1.5.0) on a VisualSonics workstation according to the manufacturer’s manual. After ventricular boundaries were outlined, the speckle-tracking algorithm automatically assigned circumjacent areas adjoining to the outlined chamber. Subsequently, the software followed the defined boundary area and calculated velocity, displacement, strain and strain rate for every spot on the defined line. Strain is commonly used to describe the deformation of an object, in our case, myocardial deformation during systole. Deformation analysis including strain and strain rate allows discriminating these parts. The amount of deformation (positive or negative strain) is usually expressed in %. Strain rate describes then the rate by which the deformation occurs over time. The unit for strain rate then is s^-1^ [41, 42]. The image data was processed using two different presentation modes. In the first mode speckle-tracking data is presented in a high regional resolution in a color-coded mode similar to a heat map presenting diastolic and systolic wall motion and deformation. These data can be reconstructed by the VevoStrain Analysis software in a three-dimensional like way to achieve a better spatio-temporal impression of myocardial motion and deformation velocities. In a second mode of presentation, myocardial wall is split up in six segments and data is presented as mean of each segment. Values are presented as curves. The slope analysis delivers further information about motion and deformation development as well as segmental (a)synchronism by time-to-peak analysis.

We analyzed six segmental curves derived from slope presentation of the speckle-tracking analysis. Maximum values as well as time to peak values including velocity, displacement, strain and strain rate of each segment were analyzed and compared to values attained from sham operated controls.

### Histological analysis

Zebrafish control hearts and at 120 and 180dpi were collected and immediately embedded in Tissue-Tek medium for cryostat sectioning. Histological analyses were conducted using 10 μm cryosections. For better contrast, we used a modified AFOG-staining protocol (Bio-Optica, Milano, Italy), staining cardiac muscle cells in pink and collagen fibrils in blue.

### Statistics

An impartial person performed all analyses blinded to the sample group. Values are displayed as means ± SEM. Statistical analysis was performed using Sigma Plot—Scientific Data Analysis and Graphing Software (Systat Software Inc., San Jose, USA) and Prism (GraphPad Software, La Jolla, USA). Differences between groups were tested by one-way ANOVA with post hoc comparisons by Bonferroni’s multiple comparison test or paired or unpaired Student’s t test where appropriate. In all tests, p<0.05 was considered statistically significant and p<0.01 as highly significant.

## Results

### Conventional Echocardiography in adult zebrafish

**Fig 2:**
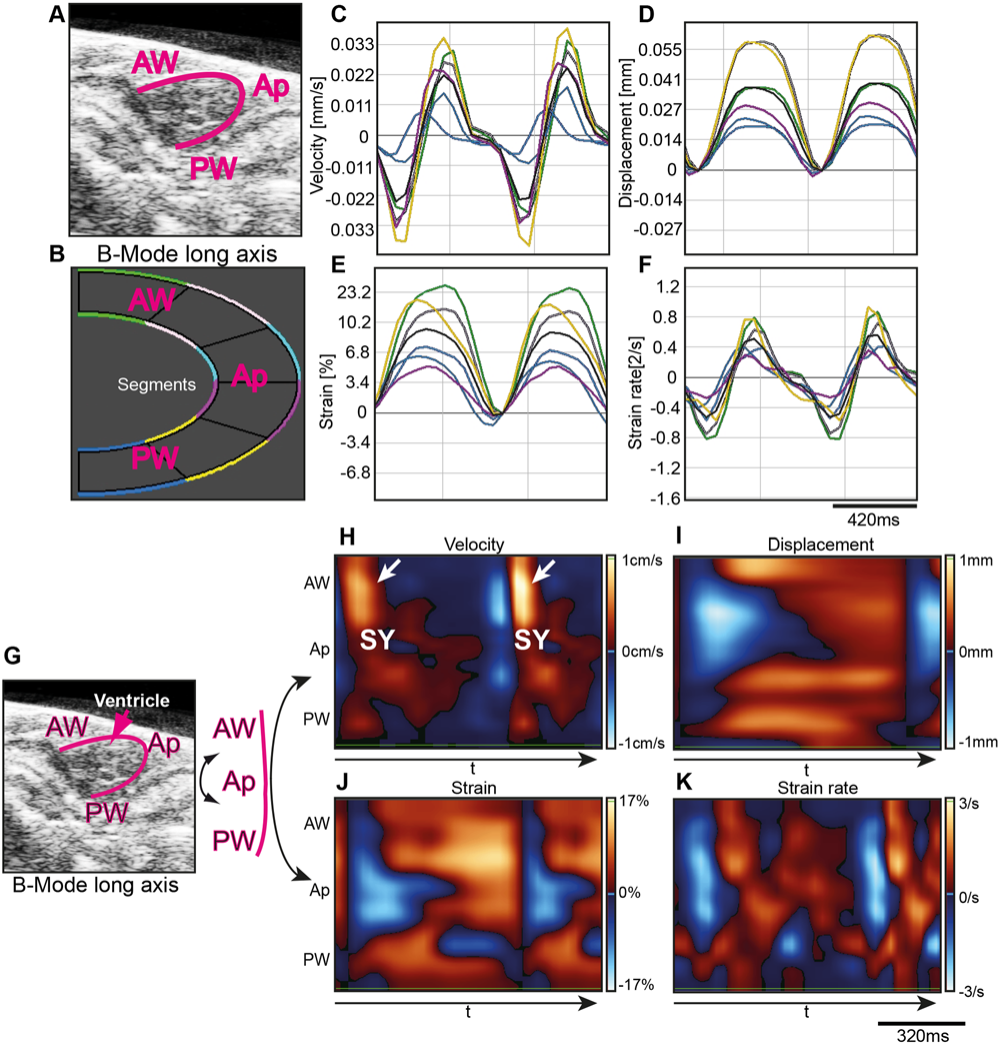
Advanced 2D wall motion in adult zebrafish hearts by speckle-tracking. (**A**) Representative LAX view with ventricular boundary outlined in pink. (**B**) Ventricle was divided into six color-coded segments as indicated with the green and light pink segment corresponding to the base of the anterior wall (AW), light blue and purple segments including the apex (Ap) and the dark blue and yellow segment corresponding the posterior wall (PW) base. (**C-F**) Representative segmental velocity traces (**C**), displacement traces (**D**), strain curves (**E**), and strain rate curves (**F**) for corresponding segments are shown. The black trace indicates the calculated average. (**G**) For high-resolution speckle-tracking representation the demarcated ventricular boundary was stretched as indicated with the top region representing the anterior wall (AW), the central region the Ap and the lower region the PW. (**H-K**) Absolute activity heat-maps of regional velocity (**H**), regional displacement (**I**), regional strain (**J**), and regional strain rate (**K**) of individual speckles during time (t) was plotted in a color coded fashion with high values shown in light red and low values in light blue as indicated. Notice that individual systoles (sy) and diastoles are distinguishable. White arrows in (**H**) indicate regions of high velocity in the AW in two distinct sy. Noticeably, AW activity is higher compared to the PW and Ap. Time scale bar as indicated. Ap, Apex; AW, anterior wall; PW, posterior wall; sy, systole.

**Fig 3:**
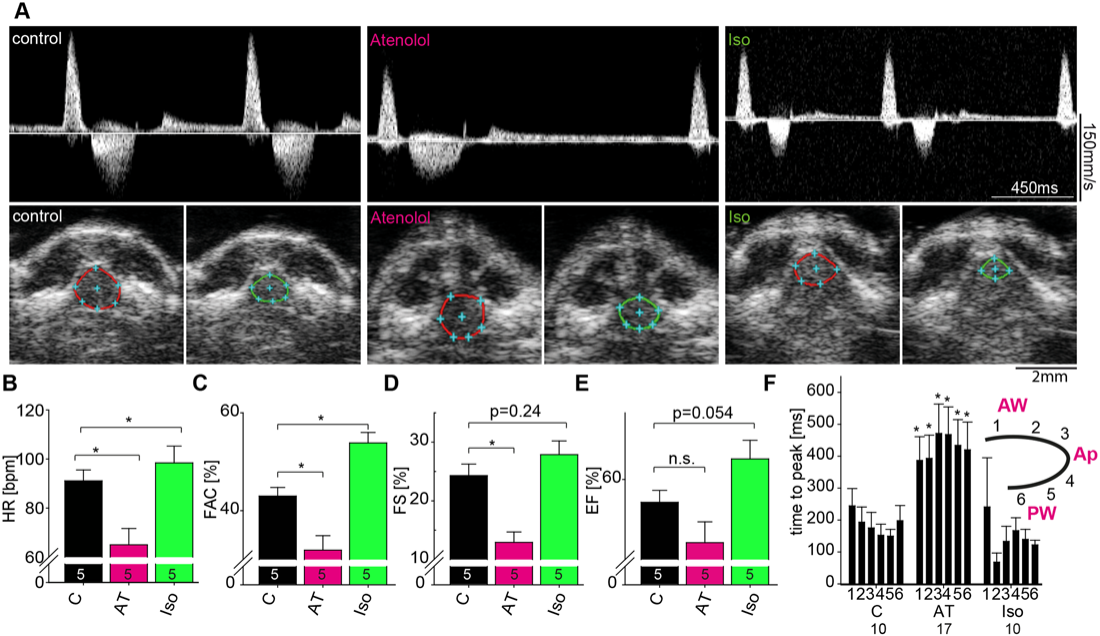
Changes in cardiac performance in response to beta-adrenergic stimulation and blockage. (**A**) Representative pulsed-wave Doppler (PWD) signals (upper row) acquired from ACX view illustrating reduced and elevated heart rate in Atenolol (middle, AT) and Isoproterenol (right, Iso) treated zebrafish, respectively, compared to controls (left). Representative SAX images (lower row) with demarcated end-diastolic (red) and end-systolic (green) ventricular dimension after Atenolol and Iso treatment. (**B-E**) Quantification of changes in heart rate (HR) (**B**), fractional area change (FAC) (**C**), fractional shortening (FS) (**D**), and ejection fraction (EF) (**E**) after AT or Iso treatment compared to controls (C). Small numbers in columns indicate number of animals measured. (**F**) For speckle-tracking analysis, the ventricle was divided in six segments as indicated with segment 1 and 2 representing the anterior wall, 3 and 4 the apex, and 5 and 6 the posterior wall. Time-to-peak analysis reveals that AT leads to significantly prolonged time-to-peak indices in myocardial velocity of all segments. Small numbers in columns indicate number of animals measured. Values are expressed as mean ± SEM. Ap, Apex; AT, Atenolol; AW, anterior wall; Iso, Isoproterenol; PW, posterior wall; *, p<0.05; unpaired student’s t-test.

The adult zebrafish heart is approximately 1000 fold smaller than a human heart and still 10 fold smaller than mice hearts. The highly trabecularized ventricle measures approximately 1mm in length and is 0.5-1mm wide, with a compact myocardial layer of 50–100μm thickness [43, 44]. To overcome this significant size limitation we deployed a high frequency ultrasound system specifically developed for small animals, with a previously demonstrated axial and lateral resolution of approximately 40μm and 70μm, respectively, hence providing the appropriate resolution for imaging [45, 46]. Importantly, this system was previously deployed for conventional echocardiography measurements in adult zebrafish [21, 24]. We initially defined three examination planes to globally measure cardiac function in adult zebrafish (Fig 1A and 1B). These three planes were specifically adapted to zebrafish anatomy and defined according to standardized echocardiographic evaluation in clinical practice. In a 60° cranial angulated transversal section, circumferential ventricular contractions can be recorded in a short axis view (SAX) using B-Mode imaging (Fig 1A1, 1B1 and 1C1, S1 Fig and Fig 1, see also S1 Movie). From these images systolic and diastolic circumferential ventricular area can be measured and the fractional area change (FAC), as a parameter for circumferential contraction, can be calculated as later shown in Fig 3A (lower row). To achieve a long axis view of the heart (LAX) we used a strictly medial craniocaudal sagittal section (Fig 1A2 and 1B2). In this view, the ventricular as well as the cardiac outflow tract (bulbus arteriosus) performance can be visualized in a sagittal long axis view (LAX) by B-Mode imaging (Fig 1C2, S2 Fig and Fig 2, see also S2 Movie). We used this imaging plane to subsequently assess the systolic and diastolic longitudinal ventricular area to calculate the ejection fraction (EF), fractional shortening (FS) and stroke volume (SV). To further characterize cardiac blood flow velocities, we applied pulsed-wave Doppler (PWD) measurements in a 45° abdominal-cranial angulated transversal plane (ACX) ensuring a parallel alignment of ultrasound beam and cardiac blood flow (Fig 1A3 and see also S3 Movie). Additionally, this plane enables visualization of the ventricular apex and the atrioventricular- and bulboventricular valves. Importantly, PWD signals have been depicted from the area of maximum inflow and outflow velocities, defined by applying C-Mode imaging. The maximum cardiac inflow velocities were usually found at the leaflets region of the atrioventricular valve, whereas the maximum outflow velocity can usually be detected, depending on the animal’s size, 35–50μm more cranial, close to the bulboventricular valve and the upper thoracic aperture. From this, a PWD signal was recorded for at least three seconds to reliably calculate the heart rate and ventricular velocity time integral (V VTI), as a parameter describing cardiac output performance (Fig 1D, see also S3 Movie). From the maximum inflow PWD signal, the heart rate, atrioventricular velocity time integral (A VTI), maximum velocity of A- and E-wave (A_max_ and E_max_) were measured and the E/A ratio, as a parameter of diastolic function was calculated (Fig 1D). The standard parameters assessed from adult zebrafish applying this protocol are presented in Table 1.

**Table 1:** Parameters attained from conventional echocardiography after the medical treatment with the beta-adrenergic therapeutics.

Measurement	Control	Atenolol	Isoproterenol
**PW-Doppler**			
Heart rate (bpm)	91 ± 4	65 ± 7	98 ± 7
E_max_ (mm/s)	32.12 ± 4.09	30.93 ± 8.27	40.93 ± 10.27
A_max_ (mm/s)	160.08 ± 12.17	155.38 ± 24.16	152.38 ± 9.66
E/A-ratio	0.21 ± 0.02	0.20 ± 0.04	0.27 ± 0.06
A VTI (mm)	7.10 ± 0.69	7.89 ± 1.60	6.50 ± 0.27
V VTI (mm)	9.24 ± 1.45	10.79 ± 2.54	12.70 ± 2.01
**Short Axis**			
Area, s (mm^2^)	0.39 ± 0.03	0.20 ± 0.03	0.37 ± 0.03
Area, d (mm^2^)	0.69 ± 0.04	0.28 ± 0.03	0.81 ± 0.09
FAC (%)	42.98 ± 1.75	31.93 ± 2.95	53.85 ± 2.14
**Long Axis**			
Area, systole (mm^2^)	0.34 ± 0.04	0.52 ± 0.10	0.35 ± 0.06
Area, diastole (mm^2^)	0.60 ± 0.06	0.78 ± 0.16	0.69 ± 0.07
FS (%)	23.52 ± 2.19	12.92 ± 1.77	27.87 ± 2.33
EF (%)	54.26 ± 2.59	45.80 ± 4.67	64.63 ± 4.19
SV (μl)	0.23 ± 0.04	0.24 ± 0.70	0.25 ± 0.04

### Advanced 2D wall motion and deformation assessment in adult zebrafish by speckle-tracking algorithms

The main aim of the study was to standardize and to advance application of echocardiography in adult zebrafish. To include speckle-tracking in adult zebrafish we initially demarcated ventricular boundaries in using B-mode images derived from LAX (Fig 2A). Importantly, still images are not suitable to accurately define chamber boundaries. Hence, we deployed B-Mode recordings of cardiac wall movements to outline systolic and diastolic ventricular dimensions (S2 Movie). Further, movement is what allows speckle-tracking to work and ultrasound generates speckle noise independent of regional resolution, enabling recording and detection of motion patterns even below resolution limits [47, 48]. After demarcating the ventricular boundary, the software then automatically defines adjacent areas to be included into the speckle-tracking analysis (see Methods section). Subsequently, regional changes for each speckle were computationally tracked and analyzed deploying speckle-tracking algorithms and velocity, displacement, strain and strain rate were calculated (Fig 2C-2K). Values for local changes showed typical, previously in mouse and humans described, curves when plotting mean values of six averaged tracked ventricular segments (Fig 2B-2F) [32, 33]. Noticeably, anterior wall segments appear to generally display slightly greater activity. Additionally, activity heat-map plotting of every individual speckle in high-resolution further provides a more detailed picture of regional myocardial motion (Fig 2G-2K). Again, activity signals derived from the anterior wall appear greater.

### Impact of beta-adrenergic manipulation on cardiac performance

Next, we wanted to assess the ability of our method to detect changes in myocardial function. Therefore, we treated adult zebrafish with the beta-adrenergic agonist Isoproterenol and the beta-adrenergic antagonist Atenolol and applied echocardiography to detect changes in chronotropy and inotropy. Treating zebrafish with 100μM Atenolol markedly reduced the heart rate as assessed by PWD (Fig 3A and 3B). Further, SAX and LAX derived measurements document a significant reduction in systolic function, as indicated by decreased EF, FS and FAC (Fig 3C-3E). SAX imaging clearly shows an increased systolic diameter after Atenolol treatment compared to controls (Fig 3A, lower row, middle column). When treating with Isoproterenol we found an increase in heart rate and systolic function (Fig 3A and 3B-3E). SAX imaging revealed enhanced systolic function and decreased end-diastolic diameter compared to controls (Fig 3A, lower row, right column). Interestingly, the speckle-tracking based velocity analysis revealed significant prolongation of time-to-peak parameters globally when treating with Atenolol, but no changes were detected when treating with Isoproterenol (Fig 3F). All basic parameters as well as speckle-tracking measurements for adult zebrafish under normal physiological conditions and under beta-adrenergic stimulation and blockage are summarized in Table 1 and S1. Hence, our protocol enables accurate capture of changes in chronotropy and inotropy after pharmacological treatment.

### Cardiac performance after cryoinjury and during heart regeneration

**Fig 4:**
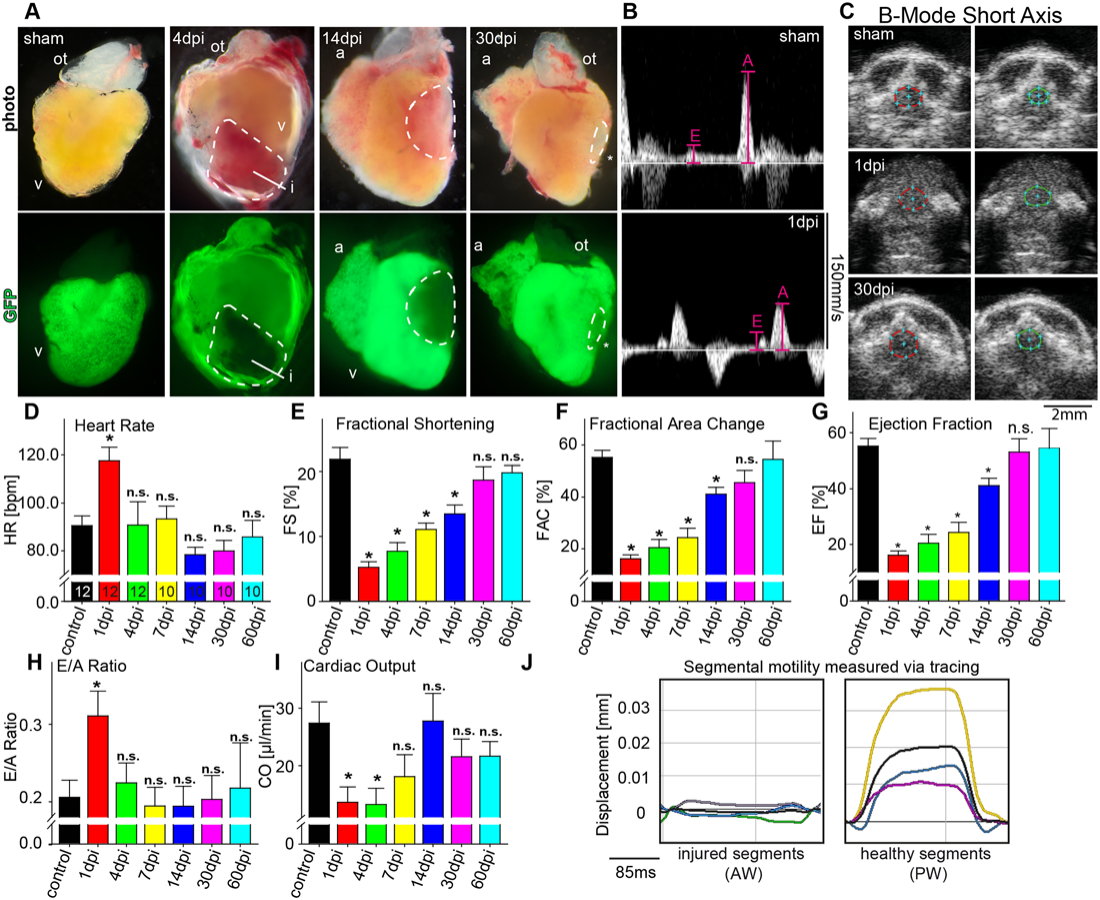
Longitudinal echocardiographic evaluation of cardiac function after cryoinjury. (**A**) Lateral brightfield (top) and fluorescent (bottom) images of hearts derived from sham operated transgenic zebrafish [*Tg(myl7*:*GFP)* ^*f1*^] and after cryoinjury at depicted time points. Dashed lines indicate injured myocardial area (i). (**B**) PWD recordings from sham (top) and cryoinjured zebrafish at 1dpi (bottom) demonstrating decreased A-wave and increased E-wave amplitudes indicative for diastolic dysfunction. (**C**) Representative SAX images from sham (upper row) and cryoinjured zebrafish at 1dpi (middle row) and at 30dpi (lower row) with end-diastolic dimensions illustrated in red and end-systolic dimensions in green. (**D-I**) Quantification of changes in (**D**) heart rate (HR), (**E**) fractional shortening (FS), (**F**) fractional area change (FAC), (**G**) ejection fraction (EF), (**H**) E/A ratio and (**I**) cardiac output (CO) at baseline and at indicated time points during regeneration after myocardial injury. Small number in (D) indicates number of animals analyzed (**J**) Speckle-tracking analysis of segmental displacement shows akinesia of injured (green, pink and light blue line) as compared to the non-injured segments (yellow, purple and dark blue line). The average curve of all segments is illustrated in black. For color coding of different segments see Fig 2B. Values are expressed as means ± SEM; a, atrium; i, injured area; ot, outflow tract; v, ventricle; AW, anterior wall; PW, posterior wall; *, p<0.05; unpaired student’s t-test and ANOVA with post hoc comparisons by Bonferroni’s multiple comparison test.

To evaluate our protocol and methodology under disease relevant conditions, we next induced myocardial necrosis in adult zebrafish by application of myocardial cryoinjury as a model of myocardial infarction [28–30]. We used a transgenic zebrafish line expressing green fluorescence protein (GFP) in cardiomyocytes [*Tg(myl7*:*GFP)* ^*f1*^] to visualize cardiomyocyte loss in response to cryoinjury and to follow regeneration (Fig 4A).

Several investigators using ex vivo molecular methodologies, predominantly supported by histology, have repeatedly shown cellular and structural processes involved in regeneration, but detailed assessment of cardiac performance during myocardial regeneration using a combination of conventional echocardiography and modern speckle-tracking based analysis has not yet been described. We performed longitudinal echocardiographic follow-up examinations of sham operated adult zebrafish at baseline condition and of cryoinjured animals at multiple time points during cardiac healing. FAC measurements from the SAX view revealed a significant reduction in circumferential contractility from 1dpi up to 14dpi (Fig 4C and 4D-4I). By 30dpi, FAC determined function was recovered. LAX view measurements showed marked decline in EF and FS beginning at 1dpi with recovery until 30dpi. SV was significantly decreased at 1dpi with subsequent continuous convergence to baseline levels until 14dpi (S2 Table, S2 Fig). Calculation of cardiac output (CO) revealed a significant reduction from 1dpi to 4dpi accompanied with elevated heart rate at 1dpi, suggestive for acute heart failure at 1dpi (Fig 4I). Additionally, diastolic function, as assessed by E/A ratio measurements derived from PWD, was dramatically affected transiently at 1dpi with complete recovery by 4dpi (Fig 4B and 4H, see also S2A and S2B Fig).

Speckle-tracking based analysis has recently been shown to be useful in detection of regional contraction abnormalities in humans and murine models of cardiac dysfunction [31, 35, 49]. Hence, we applied this new technology on adult zebrafish that underwent cryo-injury in order to attain detailed information about myocardial motion and deformation mechanics during cardiac regeneration.

**Fig 5:**
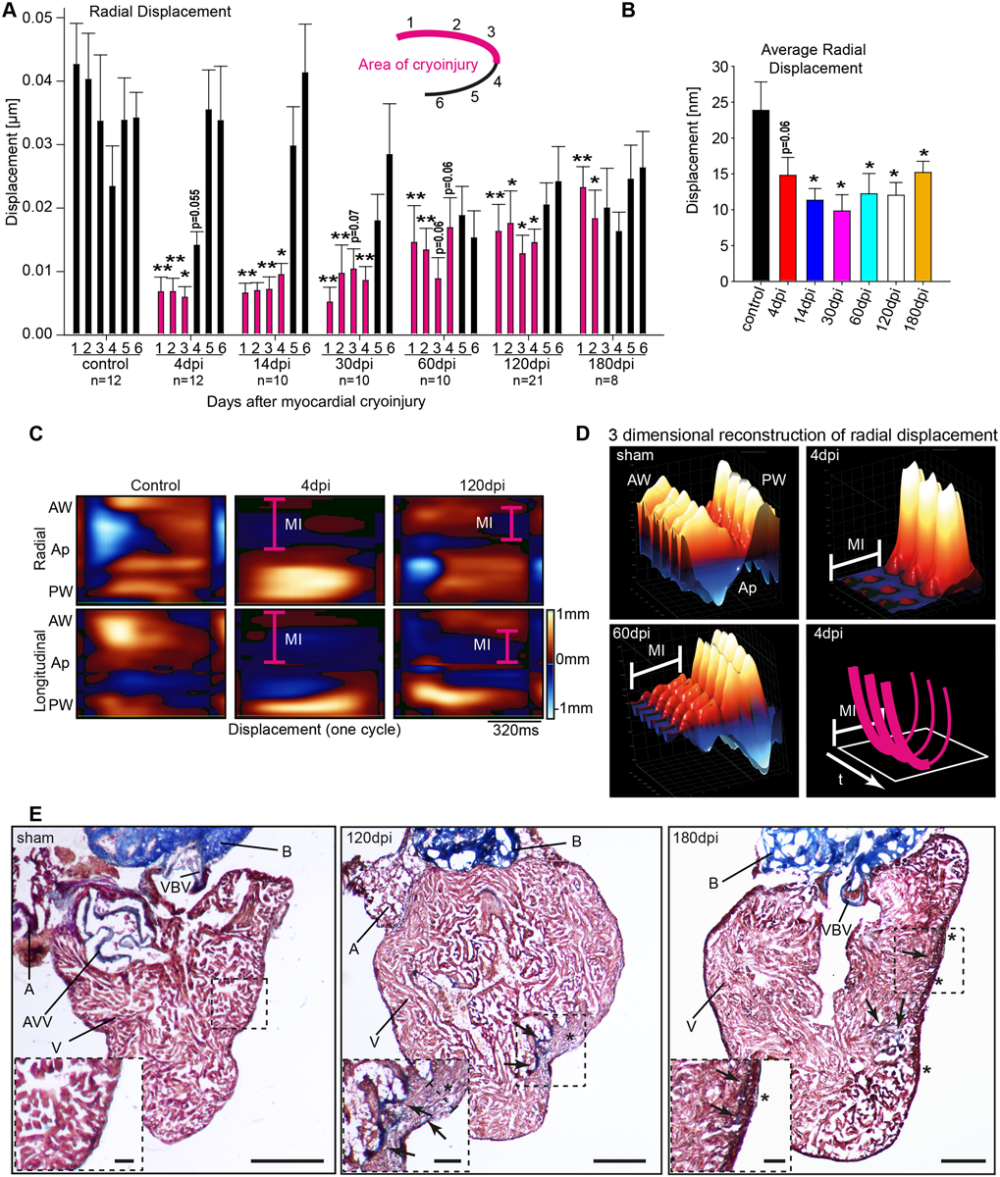
Advanced 2D wall motion measurement by speckle-tracking analysis after cryoinjury reveals segmental and regional motion and deformation disturbances. (**A**) Absolute radial displacement of individual segments at indicated time points. The ventricle was divided in six segments as indicated with segment 1 and 2 representing the anterior wall (AW), 3 and 4 the apex (Ap), and 5 and 6 the posterior wall (PW) for subsequent displacement analysis. Small numbers indicate number of animals measured. (**B**) Average of radial displacement at indicated time points. (**C**) High resolution speckle-tracking analysis of radial (upper row) and longitudinal (lower row) displacement. The top region displays displacement of the AW, the central region of the Ap and the lower region of the PW. Absolute values are color coded with high values in light red and low values in light blue as indicated. Time scale and color coding bar as indicated. The pink line at 4dpi depicts injured area (MI). (**D**) 3D reconstruction of regional displacement at indicated time points enables identification of akinesis of injured AW at 4dpi and residual wall motion deficiencies at 60dpi, respectively. The lower right image shows a schematic illustration of the 3D-reconstrations. The u-shaped pink lines indicate consecutive systoles; t and the arrow below indicate the time progress and MI the infarcted area, also indicated by the bold pink line. (**E**) Modified AFOG-staining (myocardium in red, connective tissue and fibrotic areas in blue) stained sections of a sham operated control heart and at 120 and 180dpi. AW is to the right, PW to the left. Boxed area of the cryo-injured region is shown in higher magnification in the lower left corner of its respective overview picture. At 120dpi residual fibrotic deposition (arrows) together with a thickening of the compact myocardial layer (*) can be detected. Residues of fibrosis are still detectable at 180dpi. Note that the thickening of the compact myocardial layer (*) extends over a great segment of the AW. Ap, apex; A, atrium, AVV, atrio-ventricular valve; AW, anterior wall; B, bulbus arteriosus; MI, myocardial injury; PW, posterior wall; V, ventricle; VBV, ventriculo-bulbar valve. Values are expressed as means ± SEM; *, p<0.05; **, p<0.01, unpaired student’s t-test and ANOVA with post hoc comparisons by Bonferroni’s multiple comparison test

Speckle-tracking analysis in zebrafish after cryoinjury to the anterior wall revealed markedly reduced average displacement in the affected segments of the anterior cardiac wall region, enabling clear delineation of the infarcted area to the anterior wall (Fig 4J, see also S4, S5 Movie). Particularly, segment 1 to 4 showed significantly decreased radial displacement of as much as 30dpi, while segments 1 and 2 remained significantly impaired until 180dpi (Fig 5A and 5B). Importantly, from 120dpi onwards average displacement of the injured anterior wall’s segments show significant increase in displacement compared to the average displacement of anterior wall’s segments at 4dpi, clearly indicating functional regeneration. Individual speckle-tracking in higher tempero-spatial resolution enabled an even better demarcation of the infarcted area by speckle mapping and 3D reconstruction (Fig 5C and 5D).

Gonzalez-Rosa et al. recently described, that regeneration time as assessed by histological analysis, significantly varies, particularly when using cryocautherization [29]. To evaluate, whether the observed prolonged functional impairment could be caused by residual structural alterations, we analyzed histological sections from 120 and the extended time point of 180dpi hearts. Histological analysis revealed that at 120dpi residual fibrotic scar tissue is still detectable in some hearts (Fig 5E). Further, the images revealed that very consistently in all hearts the newly formed compact myocardial layer was noticeably thickened around the injured site (Fig 5E). Surprisingly, even at 180dpi, 1 out of 8 hearts still displayed traces of fibrosis (Fig 5E). Interestingly, the condensed appearance and the thickening of the compact myocardial layer very consistently remained detectable in all analyzed hearts (n = 11) at this later time point, often covering a large portion of the AW (Fig 5E).

**Fig 6:**
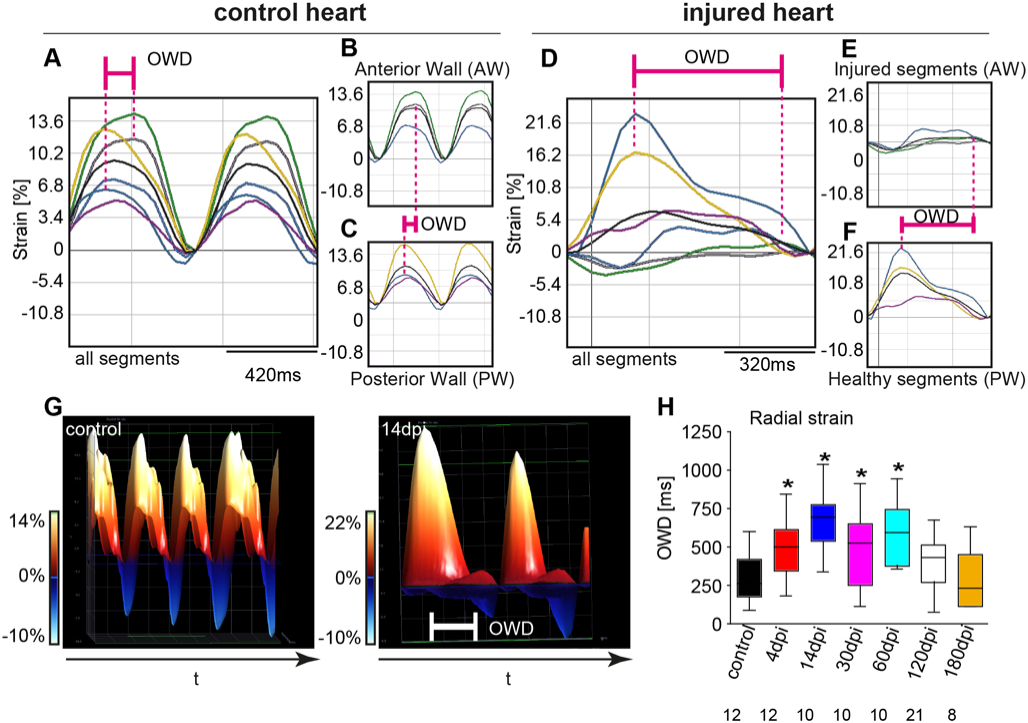
Time-to-peak and maximal opposing wall delay analysis reveals considerable asynchronicity of injured to non-injured ventricular wall. Segmental strain traces recorded from Sham. (**A-C**) and cryoinjured, regenerating hearts at 14dpi (**D-F**) are shown. (**A**) Representative segmental strain traces of non-injured hearts exhibit only minor time-to-peak delay between opposing ventricular wall segments (OWD) as illustrated by the pink line. (For color-coding explanation see Fig 2B.) (**B-C**) Individual strain curves of anterior (**B**) and posterior wall segments (**C**). (**D**) Representative segmental strain curves derived from a regenerating heart at 14dpi demonstrating considerable OWD intervals. (**E-F**) Individual strain curves of injured anterior (**E**) and non-injured posterior wall segments (**F**). For each image displayed, the black line indicates the average curve. (**G**) 3D-reconstruction of regional strain analysis derived from a control (left) and injured (right) heart at 14dpi with the frontal view rotated 90° counterclockwise. Every peak represents consecutive systoles. The anterior wall (AW) is located in the front, while the posterior wall (PW) is situated in the back. In the control presentation (left) the AW and PW peaks are completely overlapping, presenting absolute synchronicity. However, at 14dpi (right) the residual AW activity is visible as a small peak offset to the right of the PW peak in the back. This offset represents the OWD. (**H**) Quantitative analysis of mean OWDs at depicted time points. Small numbers indicate number of animals measured. Values are expressed as mean ± SEM; *, p<0.05, unpaired student’s t-test and ANOVA with post hoc comparisons by Bonferroni’s multiple comparison test.

We next assessed synchronicity of systolic anterior to posterior wall motion by investigating the time-to-peak contraction differences between fastest and slowest myocardial segment of opposite ventricular walls (opposing wall delay). As a reference point to distinguish systolic and diastolic phases during contraction, we used M-Mode recordings derived from LAX B-Mode-sequences. While in sham operated controls only minor delays between anterior and posterior wall contractions can be detected (Fig 6A-6C), the opposing wall delay after cryoinjury to the AW was significantly increased until 60dpi (Fig 6A-6H). This difference becomes even more pronounced when analyzing individual speckles in higher regional resolution and in 3D display (Fig 6G). At 14dpi asynchronism between anterior and posterior wall peaked and remained significant until 60dpi with complete recovery at least at 120dpi (Fig 6H). Detailed parameters are summarized in S3 Table.

## Discussion

Standard echocardiographic techniques were recently advanced by speckle-tracking based analysis to gain more precise information on myocardial motion and deformation characteristics with growing evidence that speckle-tracking based parameters have a greater informative value on clinical symptoms and outcome [50].

Zebrafish represent a well-established in vivo model to study cardiac development and function [1, 2, 51]. Further, zebrafish is recently used to model human cardiac disease [1–4, 9, 52, 53]. However, cardiac function in zebrafish to date was predominantly analyzed in embryonic hearts, while human cardiac dysfunction usually manifests in adult hearts. Recently, adult zebrafish have been utilized to study heart regeneration [12–18]. Novel molecular insights identified in zebrafish are highly conserved and hold translational potential possibly offering new perspectives to improve myocardial healing [13, 14, 54]. Therefore, sensitive assessment of heart function similar to mice and humans in adult zebrafish after myocardial injury is required to evaluate potential molecular intervention on organ performance.

Here, we present an advanced protocol to assess cardiac function in adult zebrafish by combining conventional echocardiography with modern speckle-tracking analysis as recently introduced in humans and mice [31, 34, 55]. We employed a standard, commercially available echocardiography device specifically designed for small animals. With this, we were able to establish the described method in approximately three months, even for a person previously not familiar in echocardiography. For a trained person, acquisition times of approximately 60–100 seconds per individual is achievable and thereby allows cardiac phenotyping of 10–100 animals in relatively short time. Importantly, every individual survives the analysis, enabling highly meaningful longitudinal studies. In our hands, when deployed on cryoinjured zebrafish, lethality did not increase and remained within the described lethality of approximately 5% [30].

Our study advances previous studies deploying conventional echocardiography by including speckle-tracking based analysis. By using high-resolution speckle-tracking we visualized and measured absolute movements in high spatial resolution. When assessing velocity, displacement and strain in the context of normal physiological conditions, we found that generally the anterior ventricular wall appear to predominantly contribute to the overall heart function. When applying cryoinjury to the anterior ventricular wall, cardiac function markedly declined. Only when anterior wall contractility recovered during regeneration, did overall heart function improve, suggesting that the posterior wall is unable to sufficiently compensate for the loss of anterior wall contractility. Within the embryonic ventricle, regional differences of the outer and the inner curvature with respect to gene expression, cell shape and function were described previously [56, 57]. Our analysis now suggests that these regional differences are conserved on a functional level till adult stages, with the anterior wall representing the former embryonic outer curvature and the posterior wall the embryonic inner curvature. Further studies are needed to confirm this observation.

Our protocol enables accurate capture of inotropic and chronotropic changes in response to pharmacological treatment. Further, previous work indicated that peak velocity indices in humans are more sensitive indicators for contraction than classical systolic measurements, particularly in detecting negative inotropy [58]. Our measurements of ventricular kinetics similarly revealed a negative dromotropic and chronotropic effect of Atenolol in adult zebrafish.

Besides many similarities to mammalian heart function as assessed by echocardiography, we also found significant differences. For example, we did not detect dramatic effects in inotropy when treating with Isoproterenol as expected from other animals, including mouse. One explanation, besides many others, could be that the ratio and distribution of the β-adrenergic receptor isoforms in the adult zebrafish heart is different than compared to mammals. Furthermore, and most obviously, E/A ratio in zebrafish is <1, whereas it is >1 in healthy humans. This is probably explained by the univentricular and uniatrial heart in zebrafish likely resulting in differences in hemodynamics.

By longitudinal echocardiography after cryoinjury, we found elevated E/A-ratio as an indicator for diastolic dysfunction as previously described after ventricular amputation [24, 26]. Evaluation of the underlying peak systolic A- and E-wave velocities in cryoinjured hearts revealed that the elevated E/A-ratio was due to an initial drop of the peak systolic A-wave velocity at 1dpi followed by a significant increase in the peak A-wave velocity from 4dpi to 14dpi, likely indicating a compensatory effect to impaired ventricular function during regeneration process by a gain in atrial function (S2A-S2B Fig). By deploying speckle-tracking, we were able to demarcate the injured region beyond 60dpi in high spatial and temporal resolution. Previous reports indicated that reconstitution of cardiac form and systolic function is mainly completed by 30dpi [16, 18, 24, 25, 30, 59]. Further, it was shown that electrical coupling of cardiomyocytes after myocardial injury is fully reconstituted between 30 and 45dpi, as assessed by optical mapping [25]. Our data indicate that regional myocardial dysfunction remains detectable far beyond 45dpi. Specifically, by measuring wall synchronicity, we show that regeneration of contraction synchronicity on an organ level extends beyond 60dpi and is fully reconstituted by 120dpi. Further, our tissue displacement analysis shows that residual functional impairment of the injured AW segments remains detectable at least until 180dpi (Fig 5E). Importantly, a functional regeneration is apparent since during the course of the analysis, tissue displacement of corresponding segments clearly recovers. Consistent with previous reports [29], we detected a noticeable thickening of the compact myocardial layer at the injury site even until 180dpi, representing the probable cause for the impaired AW motion we observed. While the previous study did similarly describe the thickening of the compact layer, they did not observe fibrotic tissue traces beyond 130dpi [29]. This discrepancy has probably technical reasons, since we used a cryoprobe significantly thicker (0.8mm; according to Chablais et al. [30]) then the one used by Gonzalez-Rosa et al. (0.3mm), likely explaining the prolonged regeneration time. This suggests that the method for induction of cryoinjury to study heart regeneration strongly influences the kinetics of the regeneration process.

So far only a few groups applied cryocautherization [28–30]. Only one study demonstrated that structural recovery time in response to cryoinjury varies and in general takes significantly longer than after ventricular resection, where complete structural recovery can be seen after 30dpi [29]. Importantly, the majority of groups applying cryoinjury did not analyze stages beyond 60dpi, although they clearly show that regeneration was not completed, supporting that the regeneration time after cryocautherization indeed extends far beyond 60dpi [28, 30]. Our study now shows that functional recovery in response to cryoinjury similarly requires dramatically longer than previous histomorphological reports suggested. We were able to identify three distinct phases of functional recovery after myocardial injury (summarized in S3 Fig). In phase (1) ending at 4dpi, diastolic function is restored and acute heart failure is compensated. The rapid restoration of diastolic function is consistent with previous reports [24]. In phase (2), at 30dpi, circular and longitudinal contractility is reinstated [24, 26]. This stage coincides with stages were structural regeneration is essentially completed [16, 18, 28, 30]. However, previous reports demonstrated that the time course for structural reconstitution varies specifically in response to cryoinjury [29]. In phase (3) lasting until 120dpi and extends beyond 180dpi, we found full reconstitution of organ synchronicity by 120dpi. Radial tissue displacement however remains impaired until and probably beyond 180dpi consistent with persisting histomorphological abnormalities of the compact layer.

The question remains, whether these residual structural alterations in the myocardial compact layer at the injury site ever normalize at later stages. Particularly since our data suggest that these alterations cause slight but detectable functional deficiencies. Future studies combining i.e. high throughput omics analysis with sensitive functional echocardiography as shown here might be used to identify signaling pathways and cellular characteristics particularly essential for the late phase of cardiac functional regeneration.

Taken together, our here described protocol is the first report that combines classical echocardiography with modern speckle tracking based analysis to assess cardiac function in adult zebrafish. It allows easy and sensitive detection of even weak and regionally distinct functional changes. This high sensitivity enabled the isolation of novel insights into the cardiac regeneration process in adult zebrafish. We believe that when systematically deployed on, i.e. existing genetic models of cardiac dysfunction, this technology can lead to novel insights in the development of cardiac disease and in heart regeneration beyond the classically used larval stages.

## Supporting Information

S1 Fig Anatomical structures in echocardiography images. Representative images of SAX view (1) and LAX view (2) acquisition indicating anatomical features in green in (1) outlining the gills (g), orange in (2) outlining the bulbus arteriosus (b), red in (1) and (2) outlining the ventricle (v), blue in (1) and (2) outlining the atrium (a), sinus venosus in (2) in yellow (sv) and artrioventricular valve in (2) in white.
(TIF)

S2 Fig E-wave and A-wave velocities and stroke volume measurements in the course of regeneration. E-wave (**A**) and A-wave (**B**) velocities, and stroke volume (**C**) at baseline and at indicated time points during regeneration after myocardial injury. Values are expressed as means ± SEM. p< 0.05; number of animals as indicated in Fig 4D.
(TIF)

S3 Fig Modern echocardiography can distinguish between 3 phases of functional regeneration. Schematic view on the time course of functional recovery after cryoinjury (day 0) as known from the literature and from our study. Phase 1 represents acute diastolic (E/A ratio) dysfunction, increased heart rate and systolic dysfunction (stroke volume) (until day 4 after cryoinjury). In Phase 2 global systolic parameters are normalized whereas distinct regions are still not completely recovered (until day 30 after cryoinjury). In Phase 3 (extends up to 120 days after cryoinjury) organ synchronicity recovers by 120dpi, however regional contractility together with histomorphological abnormalities remain detectable beyond 180dpi. Based on the literature and beside our functional data, we included other characteristics of the regenerative process above the timeline [24–26].
(TIF)

S1 Table Parameters attained from the speckle-tracking algorithm after medical treatment with the beta-adrenergic therapeutics. (DOCX)

S2 Table Parameters attained from conventional echocardiography after myocardial cryo-injury (DOCX)

S3 Table Parameters attained from the speckle-tracking algorithm after myocardial cryoinjury. (DOCX)

S1 Movie A representative short axis view of a non-injured heart presenting the round-shaped ventricle with its circular contraction. (MOV)

S2 Movie A representative long axis view of a non-injured heart. (MOV)

S3 Movie A representative pulsed-wave Doppler recording of a non-injured heart. (MOV)

S4 Movie Representative long axis view of a non-injured heart. The dynamic vectors assigned by the speckle-tracking algorithm visualize amplitude and direction of myocardial wall displacement.
(MOV)

S5 Movie Representative long axis view of a cryoinjured heart at 4dpi. The dynamic vectors assigned by the speckle-tracking algorithm clearly show highly reduced displacement at the injured anterior cardiac wall.
(MOV)
