# Supplementary figures and images for: Advanced Echocardiography in Adult Zebrafish Reveals Delayed Recovery of Heart Function after Myocardial Cryoinjury
Source: PLoS One. 2015 Apr 8;10(4):e0122665. doi: 10.1371/journal.pone.0122665 (PMC4390243; doi:10.1371/journal.pone.0122665)

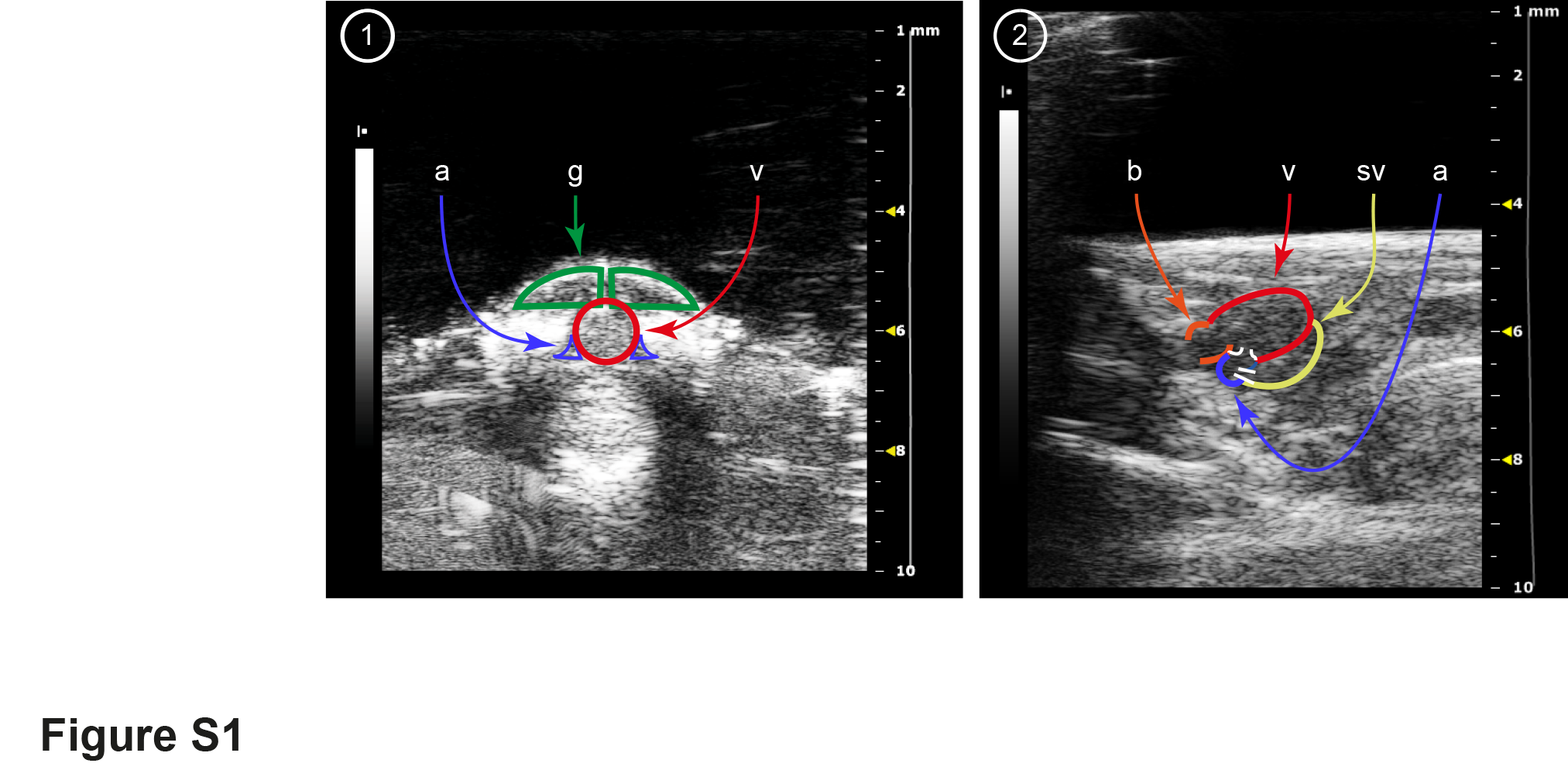

Supplement: S1 Fig — Representative images of SAX view (1) and LAX view (2) acquisition indicating anatomical features in green in (1) outlining the gills (g), orange in (2) outlining the bulbus arteriosus (b), red in (1) and (2) outlining the ventricle (v), blue in (1) and (2) outlining the atrium (a), sinus venosus in (2) in yellow (sv) and artrioventricular valve in (2) in white. (TIF) [file pone.0122665.s001.tif]

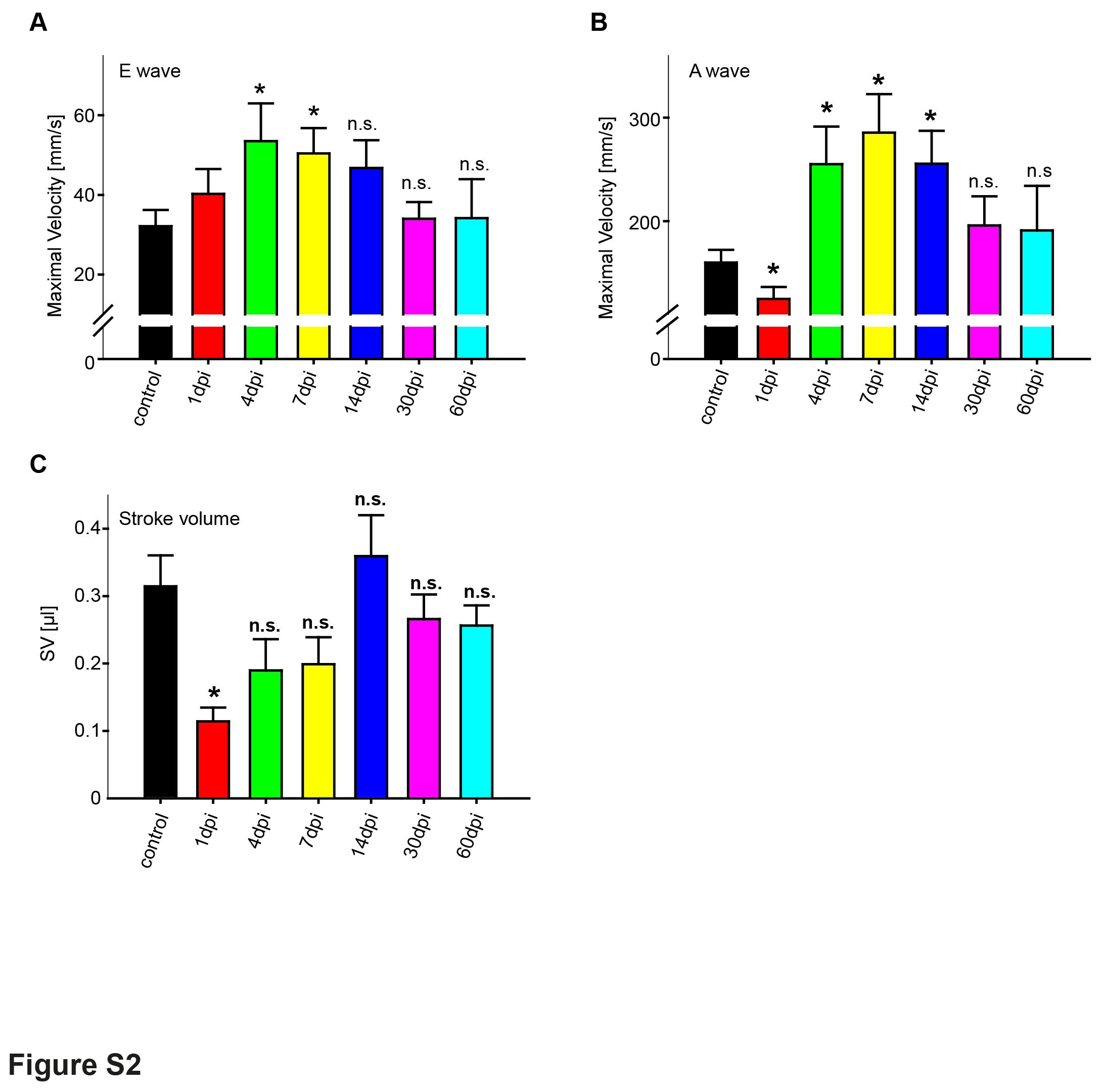

Supplement: S2 Fig — E-wave (A) and A-wave (B) velocities, and stroke volume (C) at baseline and at indicated time points during regeneration after myocardial injury. Values are expressed as means ± SEM. p< 0.05; number of animals as indicated in Fig 4D. (TIF) [file pone.0122665.s002.tif]

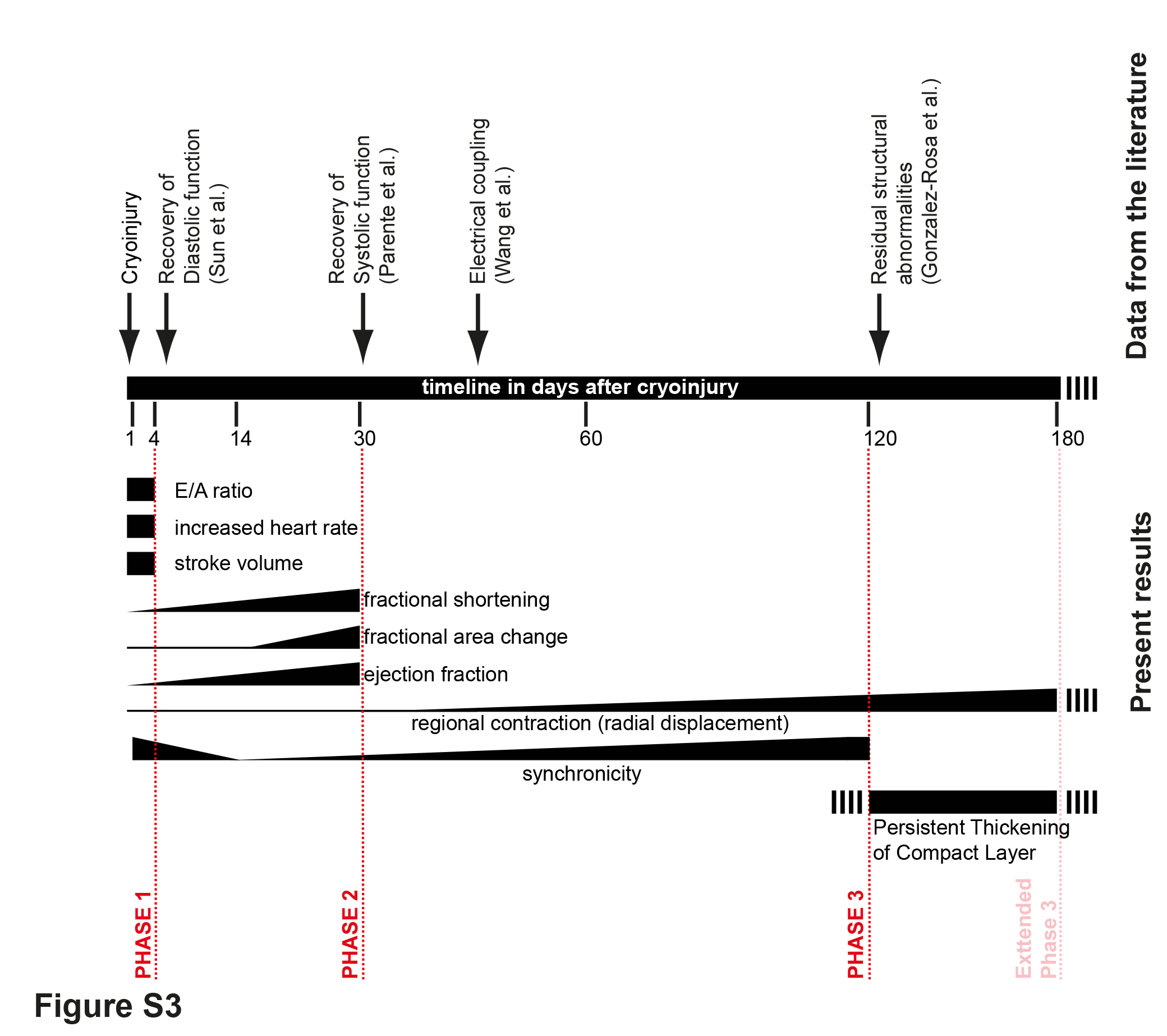

Supplement: S3 Fig — Schematic view on the time course of functional recovery after cryoinjury (day 0) as known from the literature and from our study. Phase 1 represents acute diastolic (E/A ratio) dysfunction, increased heart rate and systolic dysfunction (stroke volume) (until day 4 after cryoinjury). In Phase 2 global systolic parameters are normalized whereas distinct regions are still not completely recovered (until day 30 after cryoinjury). In Phase 3 (extends up to 120 days after cryoinjury) organ synchronicity recovers by 120dpi, however regional contractility together with histomorphological abnormalities remain detectable beyond 180dpi. Based on the literature and beside our functional data, we included other characteristics of the regenerative process above the timeline [24–26]. (TIF) [file pone.0122665.s003.tif]
