# Supplementary material for: Advanced Echocardiography in Adult Zebrafish Reveals Delayed Recovery of Heart Function after Myocardial Cryoinjury
Source: PLoS One. 2015 Apr 8;10(4):e0122665. doi: 10.1371/journal.pone.0122665 (PMC4390243; doi:10.1371/journal.pone.0122665)
Supplement: S1 Table — (DOCX) [file pone.0122665.s004.docx]

| Measurement | control | Atenolol | Isoproterenol |
| --- | --- | --- | --- |
| Average Radial Speckle-Tracking Parameters | | | |
| Velocity (µm/s) | 0.030 ± 0.011 | 0.023 ± 0.0047 | 0.17 ± 0.0049 |
| Displacement (µm) | 0.018 ± 0.004 | 0.028 ± 0.0065 | 0.020 ± 0.0045 |
| Strain (%) | 5.23 ± 1.24 | 6.17 ± 1.95 | 2.51 ±1.59 |
| Strain rate (1/s) | 0.68 ± 0.14 | 0.60 ± 0.12 | 0.47 ± 0.15 |
|  |  |  |  |
| Average Longitudinal Speckle-Tracking Parameters | | | |
| Velocity (µm/s) | 0.026 ± 0.0064 | 0.028 ± 0.0057 | 0.036 ± 0.0068 |
| Displacement (µm) | 0.024 ± 0.0060 | 0.027 ± 0.0041 | 0.023 ± 0.0090 |
| Strain (%) | 1.86 ± 0.39 | 1.79 ± 0.42 | 1.39 ± 0.60 |
| Strain rate (1/s) | 0.40 ± 0.064 | 0.40 ± 0.068 | 0.41 ± 0.07 |
|  |  |  |  |
| Radial Opposing Wall Delay (OWD) | | | |
| OWD velocity (ms) | 240.8 ± 87.85 | 427.5 ± 99.34 | 420.6 ± 159.45 |
| OWD displacement (ms) | 305.6 ± 41.08 | 470.1 ± 149.08 | 508.6 ± 132.98 |
| OWD strain (ms) | 365.4 ± 26.60 | 556.75 ± 214.65 | 618.2 ± 102.27 |
| OWD strain rate (ms) | 406.6 ± 82.72 | 689.1 ± 198.8 | 515.8 ± 99.66 |
|  |  |  |  |
| Longitudinal Opposing Wall Delay (OWD) | | | |
| OWD velocity (ms) | 369.0 ± 58.51 | 333.0 ± 93.4 | 502.8 ± 136.75 |
| OWD displacement (ms) | 284.0 ± 63.9 | 408.8 ± 111.98 | 427.8 ± 132.12 |
| OWD strain (ms) | 481.4 ± 54.08 | 658.4 ± 198.69 | 487.0 ± 89.34 |
| OWD strain rate (ms) | 420.8 ± 83.37 | 596.8 ± 110.66 | 577.8 ± 135.70 |

**Table S1**
