# Supplementary material for: Advanced Echocardiography in Adult Zebrafish Reveals Delayed Recovery of Heart Function after Myocardial Cryoinjury
Source: PLoS One. 2015 Apr 8;10(4):e0122665. doi: 10.1371/journal.pone.0122665 (PMC4390243; doi:10.1371/journal.pone.0122665)
Supplement: S2 Table — (DOCX) [file pone.0122665.s005.docx]

| Measure-ment | control | 1dpi | 4dpi | 7dpi | 14dpi | 30dpi | 60dpi |
| --- | --- | --- | --- | --- | --- | --- | --- |
| PW-Doppler | | | | | | | |
| Heart rate (bpm) | 91 ± 4 | 118 ± 6 | 91 ± 10 | 93 ± 5 | 77 ± 5 | 80 ± 4 | 86 ± 7 |
| E_max_ (mm/s) | 32.12 ± 4.09 | 40.25 ± 6.26 | 53.50 ± 9.50 | 50.42 ± 6.34 | 46.75 ± 6.98 | 34.00 ±  4.19 | 34.15 ± 9.78 |
| A_max_ (mm/s) | 160.08 ± 12.17 | 124.82 ± 11.68 | 255.10  ± 36.24 | 285.60  ± 37.15 | 255.54 ± 31.81 | 195.96  ± 28.00 | 191.00  ± 43.13 |
| E/A-ratio | 0.21 ± 0.02 | 0.31 ± 0.03 | 0.22 ± 0.03 | 0.19 ± 0.02 | 0.19 ± 0.03 | 0.20 ± 0.03 | 0.22 ± 0.06 |
| A VTI (mm) | 7.10 ± 0.69 | 6.05 ± 0.75 | 9.48 ± 1.29 | 12.30 ± 2.82 | 11.21 ± 1.19 | 8.09 ±  0.99 | 8.06 ± 1.93 |
| V VTI (mm) | 9.25 ± 1.45 | 8.32 ± 1.21 | 11.93 ± 1.63 | 12.42 ± 1.82 | 14.32 ± 1.34 | 11.09 ±  2.14 | 13.80 ± 1.64 |
|  |  |  |  |  |  |  |  |
| Short Axis | | | | | | | |
| Area, s (mm²) | 0.34 ± 0.02 | 0.68 ± 0.09 | 0.72 ± 0.11 | 0.59 ± 0.07 | 0.48 ± 0.04 | 0.35±  0.03 | 0.21 ± 0.03 |
| Area, d (mm²) | 0.62 ± 0.03 | 0.83 ± 0.11 | 0.85 ± 0.13 | 0.72 ± 0.08 | 0.65 ± 0.05 | 0.51 ±  0.03 | 0.38 ± 0.05 |
| FAC (%) | 44.54 ± 1.50 | 18.29 ± 1.37 | 15.96 ± 1.20 | 18.55 ± 0.87 | 27.18 ± 1.61 | 34.50 ±  3.36 | 46.47 ± 2.17 |
|  |  |  |  |  |  |  |  |
| Long Axis | | | | | | | |
| Area, s (mm²) | 0.52 ± 0.07 | 0.79 ± 0.04 | 0.96 ± 0.09 | 0.92 ± 0.10 | 0.84 ± 0.07 | 0.63 ±  0.05 | 0.44 ± 0.08 |
| Area, d (mm²) | 1.00 ± 0.10 | 0.91 ± 0.05 | 1.13 ± 0.11 | 1.13 ± 0.12 | 1.18 ± 0.10 | 0.94 ± 0.06 | 0.73 ± 0.08 |
| FS (%) | 21.83 ± 1.80 | 5.24 ± 0.85 | 7.72 ± 1.33 | 11.07 ± 0.99 | 13.47 ± 1.36 | 18.68 ± 2.05 | 19.80 ± 1.13 |
| EF (%) | 55.19 ± 2.73 | 16.12 ± 1.52 | 20.46 ± 3.16 | 24.31 ± 3.64 | 41.15 ± 2.64 | 45.62 ±  4.70 | 54.42 ± 7.08 |
| SV (µl) | 0.31 ± 0.05 | 0.11 ± 0.02 | 0.19 ±  0.05 | 0.20 ± 0.04 | 0.36 ± 0.06 | 0.27 ±  0.04 | 0.26 ± 0.03 |
| CO (µl/min) | 27.41 ± 3.70 | 13.62 ± 2.66 | 13.23 ± 2.81 | 18.09 ± 3.82 | 27.78 ± 4.81 | 21.56 ± 3.07 | 21.65 ± 2.55 |

**Table S2**
