# Supplementary material for: Advanced Echocardiography in Adult Zebrafish Reveals Delayed Recovery of Heart Function after Myocardial Cryoinjury
Source: PLoS One. 2015 Apr 8;10(4):e0122665. doi: 10.1371/journal.pone.0122665 (PMC4390243; doi:10.1371/journal.pone.0122665)
Supplement: S3 Table — (DOCX) [file pone.0122665.s006.docx]

| Measure-ment | control | 4dpi | 14dpi | 30dpi | 60dpi | 120dpi | 180 dpi |
| --- | --- | --- | --- | --- | --- | --- | --- |
| Average Radial Speckle-Tracking Parameters | | | | | | |  |
| Velocity (µm/s) | 0.027 ± 0.0065 | 0.016 ± 0.0025 | 0.022 ± 0.0054 | 0.011 ± 0.0049 | 0.011 ± 0.0024 | 0.017 ± 0.0023 | 0.012 ± 0.0022 |
| Displacement (µm) | 0.063 ± 0.0049 | 0.015 ± 0.0025 | 0.011 ± 0.0016 | 0.010 ± 0.0023 | 0.012 ± 0.0028 | 0.012 ±  0.0018 | 0.036 ±  0.0016 |
| Strain (%) | 3.67 ± 1.75 | 4.10 ± 0.49 | 3.71  ± 0.58 | 3.14  ± 0.70 | 3.01 ± 0.65 | 5.40  ± 1.25 | 7.97  ± 3.53 |
| Strain rate (1/s) | 0.34 ± 0.10 | 0.48 ± 0.12 | 1.07 ± 0.24 | 0.36 ± 0.07 | 0.32 ± 0.07 | 0.60 ± 0.069 | 0.64 ± 0.25 |
|  |  |  |  |  |  |  |  |
| Average Longitudinal Speckle-Tracking Parameters | | | | | | |  |
| Velocity (µm/s) | 0.020 ± 0.0033 | 0.018 ± 0.0019 | 0.020 ± 0.0039 | 0.012 ± 0.0043 | 0.016 ± 0.0027 | 0.021 ±  0.0029 | 0.022 ±  0.0036 |
| Displacement (µm) | 0.008 ± 0.0030 | 0.012 ± 0.0027 | 0.023 ± 0.0060 | 0.014 ± 0.0040 | 0.016 ± 0.0031 | 0.017 ±  0.0020 | 0.024 ±  0.0058 |
| Strain (%) | 1.99 ± 1.45 | 1.50 ± 0.49 | 3.25 ± 1.05 | 1.94 ± 0.63 | 3.00 ± 1.15 | 5.20 ±  1.21 | 5.41 ±  2.06 |
| Strain rate (1/s) | 0.59 ± 0.22 | 0.21 ± 0.050 | 0.34 ± 0.064 | 0.22 ± 0.073 | 0.25 ± 0.062 | 0.50 ±  0.08 | 0.48 ±  0.01 |
|  |  |  |  |  |  |  |  |
| Radial Opposing Wall Delay (OWD) | | | | | | |  |
| OWD velocity (ms) | 364.0 ± 73.03 | 416.8 ± 78.93 | 419.8 ± 65.40 | 331.3 ± 70.40 | 417.1 ± 59.08 | 219.8 ±  26.13 | 345.0 ±  48.95 |
| OWD displacement (ms) | 314.3 ± 32.20 | 323.5 ± 49.70 | 570.4 ± 52.55 | 408.5 ± 50.91 | 458.4 ± 74.50 | 381.1 ± 33.75 | 510.0 ± 81.33 |
| OWD strain (ms) | 308.0 ± 35.71 | 499.54 ± 55.25 | 673.2 ± 49.90 | 502.8 ± 74.33 | 626.4 ± 77.08 | 416.0 ± 36.46 | 300.0 ± 72.51 |
| OWD strain rate (ms) | 431.7 ± 65.39 | 568.7± 76.99 | 670.3 ± 62.63 | 397.2 ± 93.83 | 443.1 ± 47.06 | 445.4 ± 53.90 | 603.5 ± 97.09 |
| Longitudinal OWD | | | | | | |  |
| OWD velocity (ms) | 365.05 ± 50.53 | 385.9 ± 65.70 | 534.4 ±  66.25 | 438.7 ± 87.90 | 479.0 ± 63.07 | 442.1 ±  76.02 | 455.0 ±  48.95 |
| OWD displacement (ms) | 337.5 ± 41.56 | 466.8 ± 70.94 | 553.1 ± 45.81 | 332.3 ± 67.35 | 593.6 ± 81.79 | 298.9 ± 36.62 | 492.5 ± 64.69 |
| OWD strain (ms) | 436.1 ± 33.47 | 533.2 ± 60.80 | 576.6 ±  39.28 | 526.4 ± 51.02 | 582.1 ± 70.54 | 494.9 ±  30.96 | 635.0 ±  59.25 |
| OWD strain rate (ms) | 503.8 ± 58.35 | 571.8 ± 64.34 | 580.9 ± 57.67 | 508.9 ± 55.19 | 543.7 ± 64.99 | 394.79 ± 36.51 | 609.25 ± 63.20 |

**Table S3**
